# Wilson–Cowan Equations for Neocortical Dynamics

**DOI:** 10.1186/s13408-015-0034-5

**Published:** 2016-01-04

**Authors:** Jack D. Cowan, Jeremy Neuman, Wim van Drongelen

**Affiliations:** Department of Mathematics, University of Chicago, 5734 South University Avenue, Chicago, IL 60637 USA; Department of Physics, University of Chicago, 5720 South Ellis Avenue, Chicago, IL 60637 USA; Department of Pediatrics, University of Chicago, KCBD 900 East 57th Street, Chicago, IL 60637 USA

**Keywords:** Wilson–Cowan equations, Bogdanov–Takens bifurcation, Propagating decaying LFP and VSD waves, Localized decaying LFP and VSD responses, Neural network master equation, Directed percolation phase transition, Pair-correlations

## Abstract

In 1972–1973 Wilson and Cowan introduced a mathematical model of the population dynamics of synaptically coupled excitatory and inhibitory neurons in the neocortex. The model dealt only with the mean numbers of activated and quiescent excitatory and inhibitory neurons, and said nothing about fluctuations and correlations of such activity. However, in 1997 Ohira and Cowan, and then in 2007–2009 Buice and Cowan introduced Markov models of such activity that included fluctuation and correlation effects. Here we show how both models can be used to provide a quantitative account of the population dynamics of neocortical activity.

We first describe how the Markov models account for many recent measurements of the resting or spontaneous activity of the neocortex. In particular we show that the power spectrum of large-scale neocortical activity has a Brownian motion baseline, and that the statistical structure of the random bursts of spiking activity found near the resting state indicates that such a state can be represented as a percolation process on a random graph, called *directed* percolation.

Other data indicate that resting cortex exhibits pair correlations between neighboring populations of cells, the amplitudes of which decay slowly with distance, whereas stimulated cortex exhibits pair correlations which decay rapidly with distance. Here we show how the Markov model can account for the behavior of the pair correlations.

Finally we show how the 1972–1973 Wilson–Cowan equations can account for recent data which indicates that there are at least two distinct modes of cortical responses to stimuli. In mode 1 a low intensity stimulus triggers a wave that propagates at a velocity of about 0.3 m/s, with an amplitude that decays exponentially. In mode 2 a high intensity stimulus triggers a larger response that remains local and does not propagate to neighboring regions.

## Introduction

The analysis of large-scale brain activity is a difficult problem. There are about 50 billion neurons in the cortex of the human brain: 80 % are excitatory, whereas the remaining 20 % are inhibitory. Each neuron has about seven thousand axon terminals from other neurons, but there is some redundancy in the connectivity so that it has effective connections from about 80 other neurons, mostly nearest neighbors. Each neuron is actually a complex switching device, but in this review, we introduce only the simplest cellular model, that neurons are binary switches, either quiescent or activated. It follows that there are approximately $10^{1.5 \times10^{10}}$ configurations of activated or quiescent neurons. Such a large configuration space suggests the need to use statistical methods to analyze large-scale brain activity. In addition there is some degree of microscopic randomness in neural connectivity, and there are also random fluctuations of neural activity, both of which also support the need for a statistical treatment, as noted by Sholl in 1956 [1].

## Experimental Data on Large-Scale Brain Activity

There is a large body of data on large-scale brain activity, including electroencephalographic (EEG) recordings with large electrodes from the surface of the scalp, functional magnetic resonance (fMRI) measurements of blood flow in different brain regions (also large-scale), local field potentials (LFP) recorded with smaller electrodes, microelectrode recordings from or near individual neurons, or (currently) microelectrode arrays which can record the simultaneous activity of many neighboring neurons. Currently there are also new techniques for forming optical images of local brain activity, using voltage sensitive dyes (VSD). All such recordings can be classified as either *spontaneous* or *resting* activity, or stimulus-driven *evoked* activity.

### Resting Activity

**Fig. 1:**
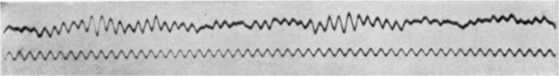
*The upper trace* is the first recording of spontaneous electrical activity from the human scalp. *The lower trace* is a 10 Hz oscillation. [Reproduced from [3]]

We first consider the resting brain activity of unanesthetized animals first observed in animals by Caton in 1875 [2], and in humans by Berger in 1924 [3]. Recordings from the human scalp are referred to as electroencephalographs (EEG) and are measured via electrodes on the unshaven scalp. The voltage differences measured between such electrode pairs are about 50 μV. Figure 1 shows a typical EEG recording.

**Fig. 2:**
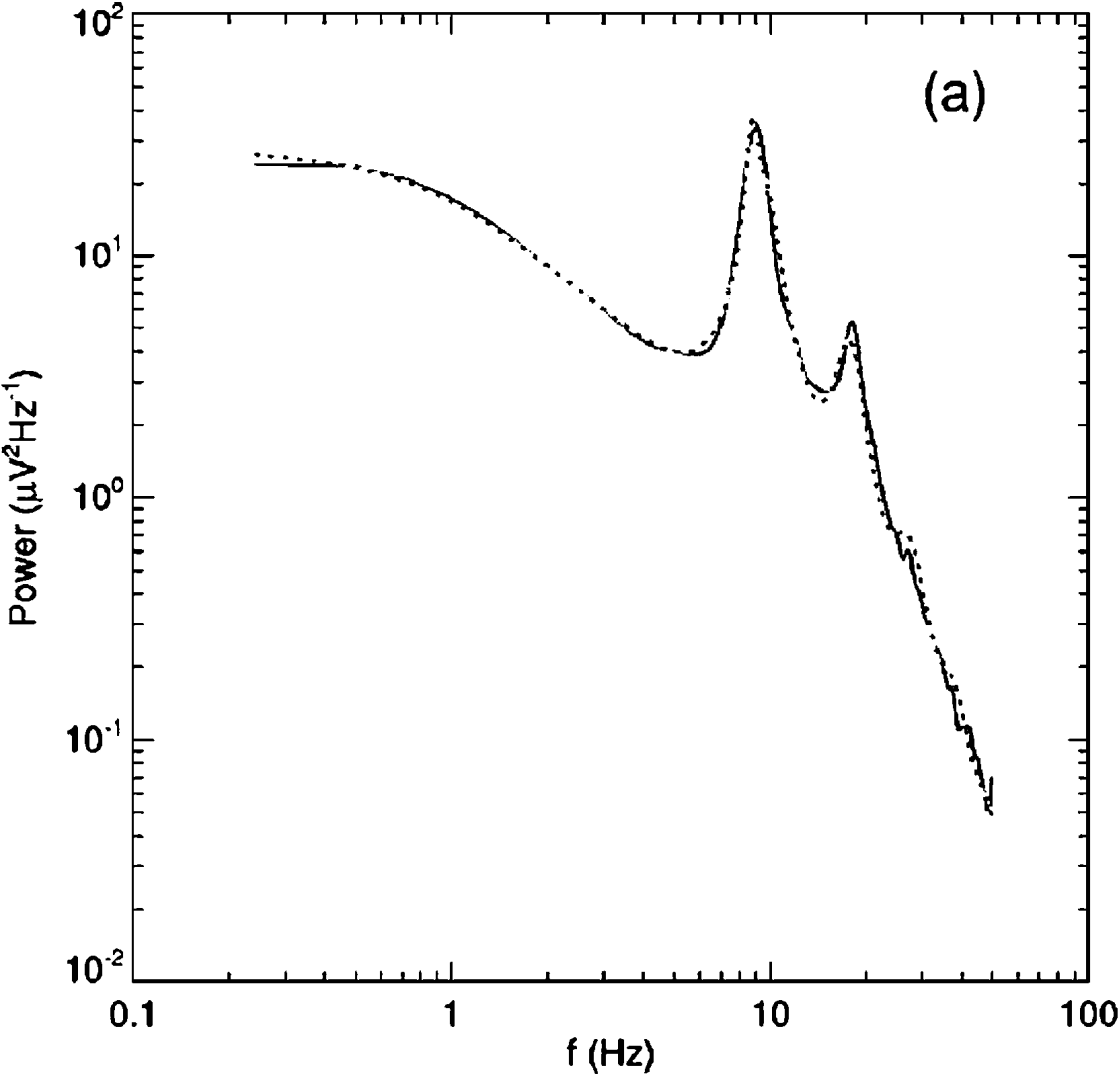
The power spectrum of the occipital EEG of a resting, awake human. [Reproduced from [4]]

**Fig. 3:**
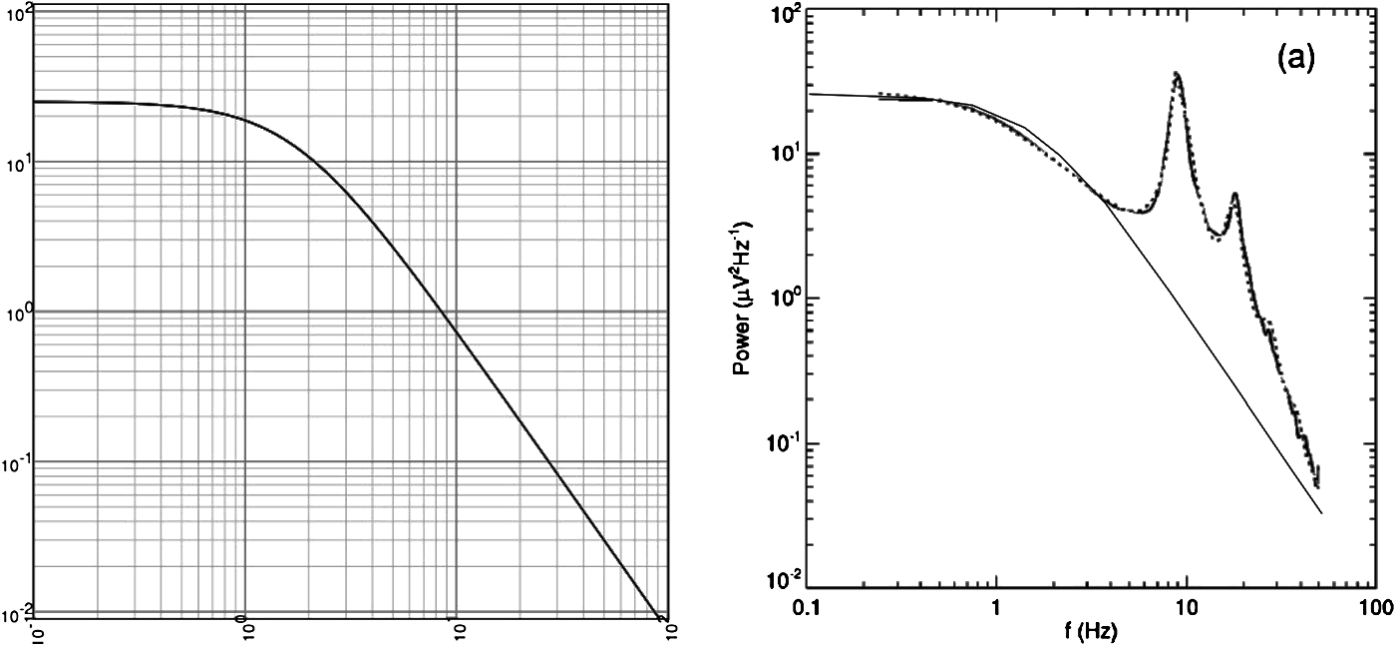
*The left panel* shows the function $75/(3+f^{2})$, *the right panel* the fit of such a function to the EEG power spectrum shown in Fig. 2

It will be seen that there are intermittent bursts of 10 Hz oscillations in the scalp activity. These oscillations comprise the *alpha* rhythm, seen in awake relaxed humans, mainly in the occipital region of the brain which processes visual signals from the eyes. Figure 2 shows the power spectrum of such activity. It will be seen that there is a pronounced peak in the power spectrum at around 10 Hz and a secondary peak around 20 Hz. This peak is said to be in the range of the *beta* rhythm of occipital EEG activity. Interestingly if the contributions of such peaks are eliminated, what is left can be fitted with the function $a/(b+f^{2})$, where *a* and *b* are constants, and *f* is the frequency in Hz. Figure 3 shows such a function and its fit to the EEG power spectrum. It is important to note that this power spectrum fit is that of Brownian motion, which suggests that resting brain activity is largely *desynchronized* and *random*.

**Fig. 4:**
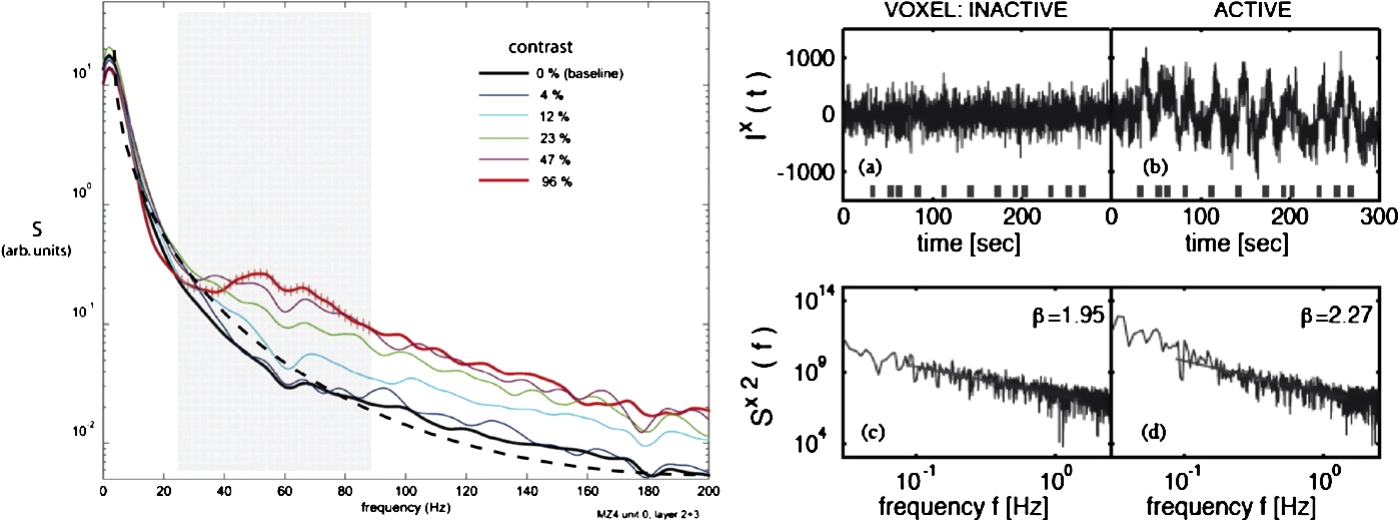
*The left panel* shows the power spectra of LFP recordings from a cat’s visual cortex in response to sine-wave modulated grating patterns. [Reproduced from [5].] *The right panel* shows fMRI recordings of both resting and stimulated human brain activity, and their associated power spectra. [Reproduced from [6]]

Other measurements of resting brain activity have been carried out on lightly anesthetized animals using local field potential recordings of spiking neuron activity, or else via fMRI measurements of blood flows in the brain that accompany unanesthetized brain activity. Figure 4 shows examples. Note the fit of the Brownian motion power spectrum $125/(5+f^{2})$ to the resting LFP.

#### Isolated Neocortex

But the most detailed studies, and the most information about the nature of spontaneous activity, has been obtained from studies of isolated neocortical slabs. The first detailed studies were carried out in the early 1950s by DeLisle Burns, on isolated slabs of parietal neocortex [7, 8]. The main relevant result was that very lightly anesthetized slabs spontaneously generated *bursts* of propagating activity from a number of randomly occurring sites. Any variation of the level of anesthesia, either up or down, abolished the activity.

**Fig. 5:**
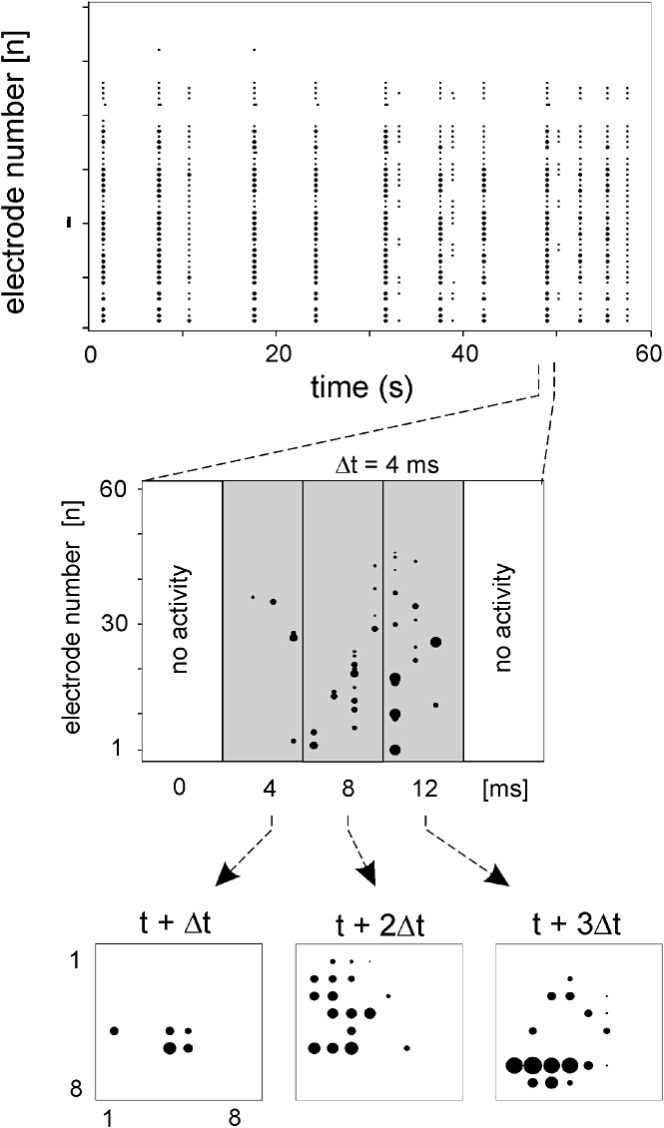
Electrode data from slices of rat neocortex. *The top graph* is a raster plot of electrode activation times. They seem synchronous, but closer examination reveals that the times exhibit self-similarity. *The bottom graphs* show a sequence of electrode activations in the original array. [Reproduced from [9]]

**Fig. 6:**
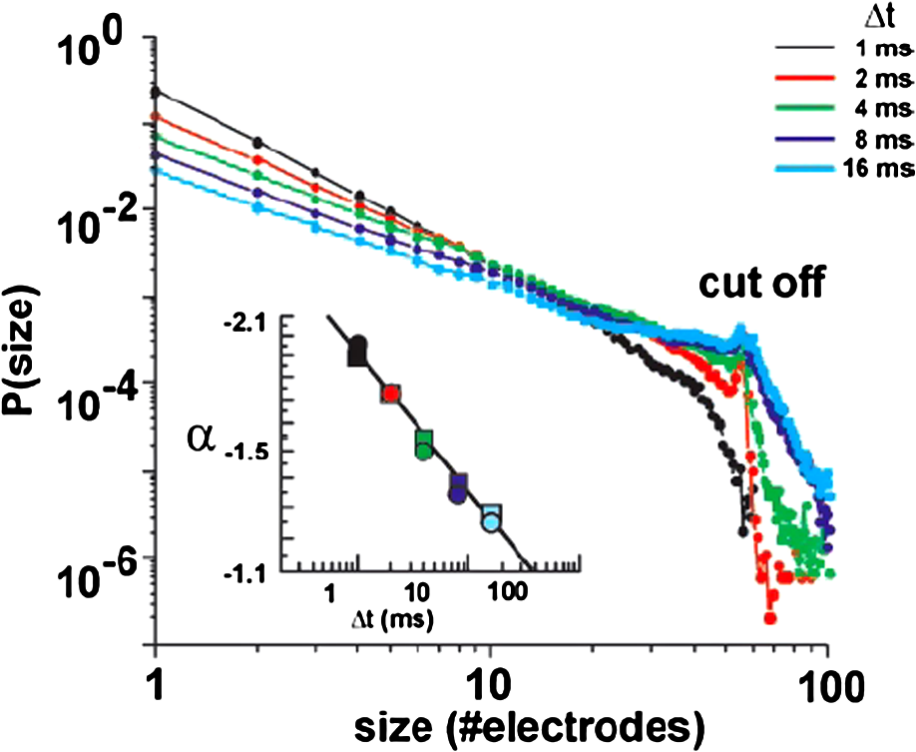
Probability distribution of burst sizes at different bin widths Δ*t*. *Inset*: Dependence of slope exponent *α* on bin width. [Reproduced from [9]]

However, it was not until 2003 that a systematic study of such burst activity was carried out by Beggs and Plenz [9] using isolated slabs of rat somatosensory cortex, either in mature tissue cultures, or else in slices. The tissue cultures exhibited spontaneous bursts of propagating activity in the form of local field potentials recorded at microelectrodes. The slices, however, were silent until stimulated with NMDA, a glutamate-receptor agonist, in combination with a dopamine $\mathrm{D_{1}}$-receptor agonist. In contrast to DeLisle Burns, Beggs and Plenz used an $8 \times8$ microelectrode array to record local field potentials (LFPs) in the slab. The main result of their experiments is summarized in Figs. 5 and 6.

Beggs and Plenz’s conclusion is that such bursts of activity are *avalanches* defined as follows: the configuration of active electrodes in the array during one time bin of width Δ*t* is termed a frame, and a sequence of frames preceded and followed by blank frames is called an avalanche. However, successive frames are not highly correlated, so the activity is not wave-like: it is in fact *self-similar*, and in addition, the avalanche size distribution follows the power law $P[n] \propto n^{\alpha}$. In addition the exponent *α* is approximately −1.5. This is the mean-field exponent of a critical branching process [10]. This result was a step beyond that of Softky and Koch [11] who found Poisson-like spiking activity in individual cortical neurons, and introduced the possibility of criticality in brain dynamics. In fact this mean-field exponent turns up in several kinds of *percolation* processes on random graphs, including both isotropic and directed percolation. But branching and annihilating random walks are equivalent to directed percolation, so it is possible that what Beggs and Plenz observed in cortical slices was a form of directed percolation. We will return to this topic later.

### Driven or Stimulated Activity

In case there is an external stimulus, neocortical dynamics indicates a very different picture. It turns out that there is a big difference in the responses to weak stimuli, compared to those triggered by stronger stimuli. In addition correlations between pairs of neurons in driven neocortex have a shorter length scale than those found in spontaneous activity.

#### Weak Stimuli

**Fig. 7:**
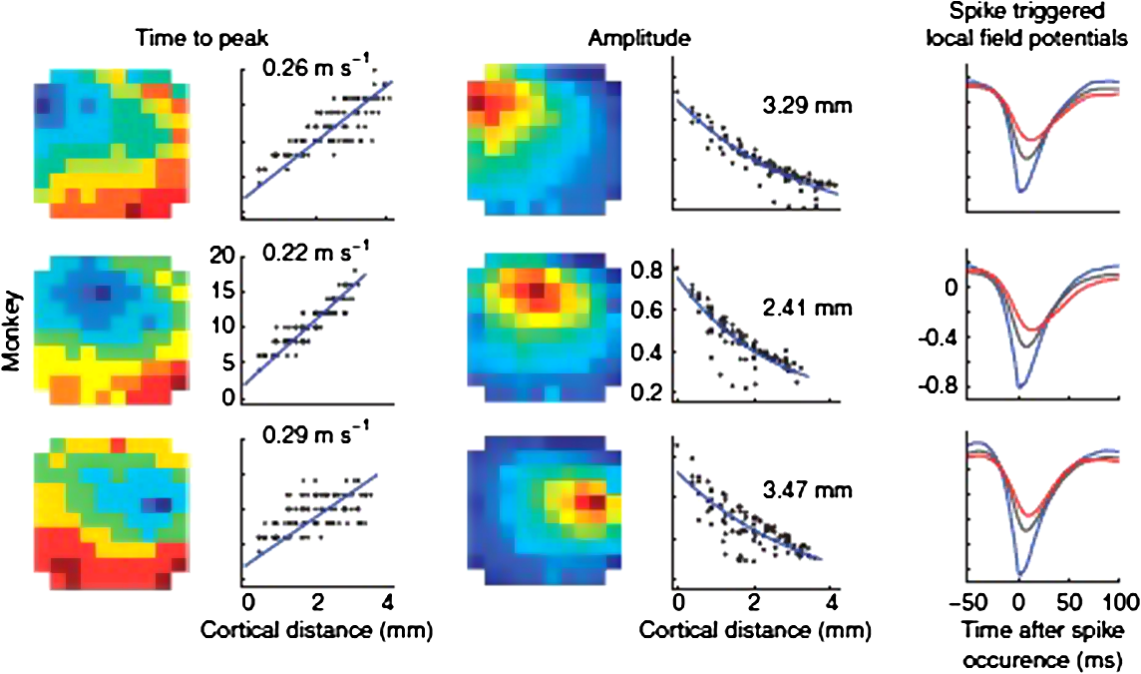
Spikes of low amplitude initiate traveling waves of LFP in the cortex. See text for details. [Reproduced from [13]]

The basic result for weak stimuli is that the cortical response is a *propagating wave* whose amplitude decays exponentially with distance. Figure 7 shows the cortical responses to low amplitude stimuli in the form of spikes, recorded by an implanted microelectrode array in three monkey visual cortices by Nauhaus et al. [13]. Each row shows data from the spike-triggered local field potentials (LFP) from a single location. The first column shows the dependence of time to peak of the LFP as a function of the cortical distance from the triggering electrode, and estimated propagation velocities. The second column shows the propagating wave, both as a pseudo-colored image, and as a plot of wave amplitude vs. distance from the triggering electrode, together with estimates of the space-constants of the decaying waves. The third column shows average LFP waveforms at three locations from the triggering spike.

It will be seen that the response is indeed a traveling LFP, whose velocity is about 25–30 cm/s. In addition the LFP amplitude decays exponentially, with a decay constant *λ* of about 3 mm.

#### Strong Stimuli

**Fig. 8:**
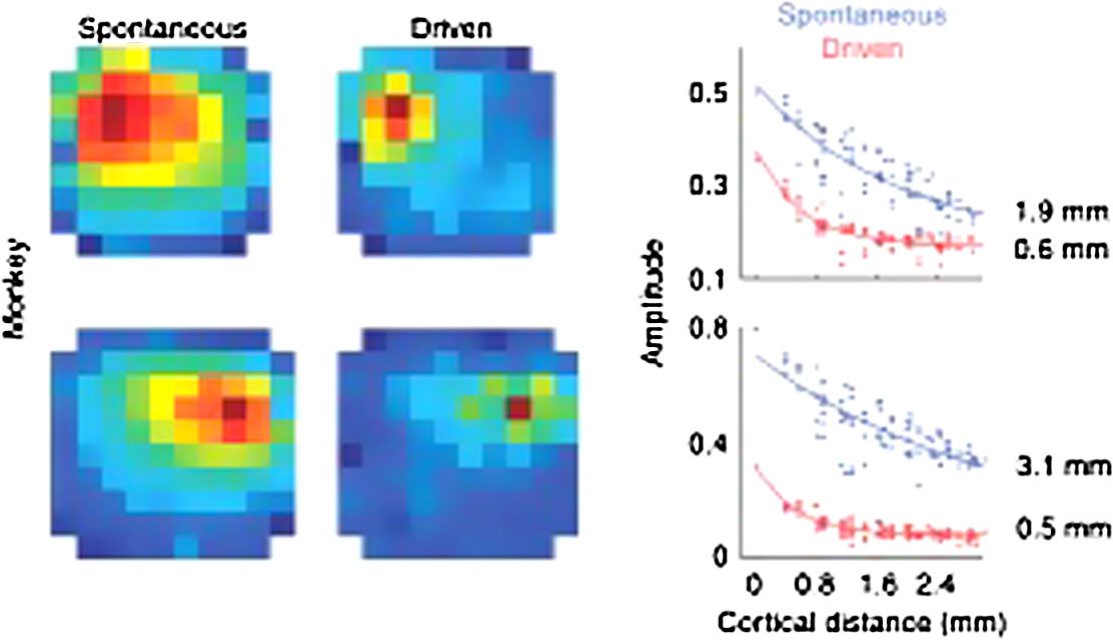
Spikes of larger amplitude initiate standing waves of LFP in the cortex. See text for details. [Reproduced from [13]]

In contrast the basic result for strong stimuli is that cortical responses to such stimuli are much more *localized*. Figure 8 shows a comparison of cortical responses to weak and strong stimuli [13]. It will be seen that responses to larger stimuli remain essentially *localized*. These observations immediately suggest a role for inhibition in localizing such responses.

#### Correlations

**Fig. 9:**
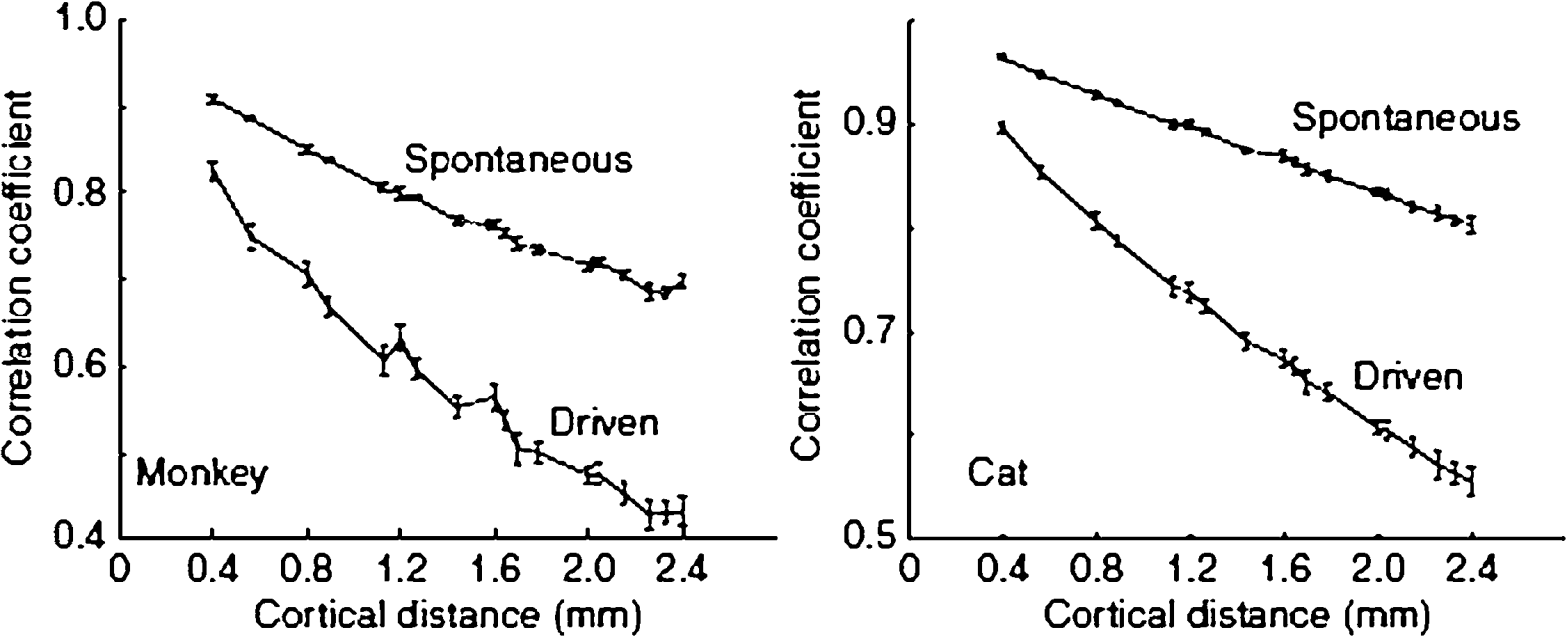
Fall of with distance of cortical pair correlations. See text for details. [Reproduced from [13]]

The basic result for correlations is that correlations between pairs of LFP fall off with separation distance, and such a falloff is much greater for strong stimuli than for weaker ones; see Fig. 9. Thus strong stimuli weaken the intrinsic pair correlations that exist in spontaneous activity. See Lampl et al. and others [16–19]. These observations also suggest a role for inhibition.

To explain all these observations we need to understand the competing roles of neural excitation and inhibition in neural population dynamics. We therefore give a short account of the history and development of the Wilson–Cowan neural population equations.

## Neural Population Equations

### Introduction

Following early work by Shimbel and Rapaport [20], Beurle [21] focused, not on the activity of single neurons, but on the proportion of neurons activated per unit time in a given volume element of a slice or slab of neocortex, denoted by $n(\mathbf {x},t)$. For all practical purposes this can be taken to be equivalent to the spike-triggered LFP and VSD described earlier.

Beurle introduced the update equation

1 $$ n(\mathbf{x}, t+\tau) = q(\mathbf{x}, t) f\bigl[n(\mathbf{x}, t)\bigr], $$

where $q(\mathbf{x}, t)$ is the density of quiescent neurons in the given volume element, and $f[n(\mathbf{x},t)]$ the proportion of neurons receiving *exactly* threshold excitation. [There is an implicit assumption that individual neurons are of the integrate-and-fire variety.]

There are three points to note here.

1. By assuming that $n( t+\tau) = q(t)f[n(t)]$ Beurle ignored the effects of fluctuations and correlations on the dynamics. It is not true that *q* and $f[n]$ are statistically independent quantities, as was first pointed out in [22].
2. The update equation is incorrect. $f[n]$ should be the proportion of neurons receiving *at least* threshold excitation, as was first noted by Uttley [23].
  This proportion can be expressed [24] as:
  2 $$ f[n] = \int_{-\infty}^{n} P(n_{\mathrm{TH}}) \,dn_{\mathrm{TH}}= \int _{-\infty}^{\infty} \vartheta[n-n_{\mathrm{TH}}]P(n_{\mathrm{TH}}) \,dn_{\mathrm{TH}}=\bigl\langle \vartheta[n] \bigr\rangle , $$
  where $\vartheta[n]$ is the Heaviside step function and $\langle \vartheta[n] \rangle$ is the average of $\vartheta[n]$ over the probability distribution of thresholds $P(n_{\mathrm{TH}})$.
  This implies that the function $f[n]$ should have the form of a probability distribution function, not a probability density. In Cowan [25] the logistic or *sigmoid* form,
  3 $$ f[n] = \bigl[1+ \exp[-n]\bigr]^{-1}=\frac{1}{2} \biggl[1+ \tanh \biggl(\frac{n}{2}\biggr)\biggr] $$
  was introduced, as an analytic approximation to the Heaviside step function used in McCulloch–Pitts neurons [26]. This indicates that the required continuum equations should represent the dynamics of a population of integrate-and-fire neurons in which there is a random distribution of thresholds.
  The corrected version of Beurle’s equation takes the form
  4 $$\begin{aligned} &n(\mathbf{x}, t+\tau) \\ &\quad= q(\mathbf{x}, t)f\bigl[n(\mathbf{x}, t)\bigr] \\ & \quad= q(\mathbf{x}, t) f \biggl[ \int_{-\infty}^{t} dt^{\prime} \int_{-\infty }^{\infty}d\mathbf{x}^{\prime}\alpha \bigl(t-t^{\prime}\bigr)\bigl[\beta\bigl(\mathbf{x}-\mathbf {x}^{\prime}\bigr)n\bigl(\mathbf{x}^{\prime}, t^{\prime}\bigr)+h\bigl(\mathbf{x},t^{\prime}\bigr)\bigr] \biggr] , \end{aligned}$$
  where
  5 $$ q(\mathbf{x}, t)=1- \int_{t-r}^{t} n(\mathbf{x},t); $$
  $r = 1\mbox{ ms}$ is the (absolute) refractory period or width of the action potential, and
  6 $$ \alpha\bigl(t-t^{\prime}\bigr)=\alpha_{0} e^{-(t-t^{\prime})/\tau},\quad\quad \beta\bigl(\mathbf {x}-\mathbf{x}^{\prime}\bigr) = b e^{-|\mathbf{x}-\mathbf{x}^{\prime}|/\sigma} $$
  are the impulse response function and spatially homogeneous weighting function of the continuum model, with membrane time constant $\tau\sim 10~\mbox{ms}$, and space constant $\sigma\sim100~\upmu \mbox{m}$.
3. Beurle’s formulation does not explicitly incorporate a role for inhibitory neurons.

### The Wilson–Cowan Equations

Wilson and Cowan corrected and extended Beurle’s work and introduced equations for the population dynamics of a spatially homogeneous population of coupled excitatory and inhibitory binary neurons [24], and its extension to spatially inhomogeneous populations [27]. These equations take the forms

7 $$ \begin{aligned} \tau\frac{dE}{dt} &= - E(t) + \bigl(1 - r E(t) \bigr) f_{E} \bigl[ w_{EE} E - w_{EI}I + h_{E}(t) \bigr] , \\ \tau\frac{dI}{dt} &= - I (t) + \bigl(1 - r I (t) \bigr) f_{I} \bigl[ w_{IE}E - w_{II} I + h_{I}(t) \bigr] , \end{aligned} $$

for the spatially homogeneous case, and

8 $$ \begin{aligned} \tau\frac{\partial E(\mathbf{x},t)}{\partial t}={}& {-}E(\mathbf{x},t) + \bigl(1-rE(\mathbf{x},t) \bigr) \\ &{}\times f_{E} \biggl[ \int_{-\infty}^{\infty}\rho_{E} \,d \mathbf{x}^{\prime}\beta _{EE} \bigl(\mathbf{x}- \mathbf{x}^{\prime}\bigr)E\bigl(\mathbf{x}^{\prime},t\bigr) \\ &{}- \int_{-\infty }^{\infty}\rho_{I}\, d \mathbf{x}^{\prime}\beta_{EI}\bigl(\mathbf{x}-\mathbf {x}^{\prime}\bigr)I\bigl(\mathbf{x}^{\prime},t\bigr)+h_{E} (\mathbf{x},t) \biggr] , \\ \tau\frac{\partial I(\mathbf{x},t)}{\partial t}={}&{-} I(\mathbf{x},t) + \bigl(1-rI(\mathbf{x},t) \bigr) \\ &{}\times f_{I} \biggl[ \int_{-\infty}^{\infty}\rho_{E} \,d \mathbf{x}^{\prime}\beta _{IE} \bigl(\mathbf{x}- \mathbf{x}^{\prime}\bigr)E\bigl(\mathbf{x}^{\prime},t\bigr)\\ &{}- \int_{-\infty }^{\infty}\rho_{I}\, d \mathbf{x}^{\prime}\beta_{II}\bigl(\mathbf{x}-\mathbf {x}^{\prime}\bigr)I\bigl(\mathbf{x}^{\prime},t\bigr)+h_{I} (\mathbf{x},t) \biggr] , \end{aligned} $$

for the continuum form of the spatial case, in which $\rho_{E}$, and $\rho _{I}$ are, respectively, the packing densities of excitatory and inhibitory cells in the cortical slab.

Note that $f_{E}[n]$ and $f_{I}[n]$ are modified versions of the firing rate function $f[n]$ introduced in Eq. (3), such that $f_{E}[0]=f_{I}[0]=0$.

Note also that the variables $E(\mathbf{x},t)$ and $I(\mathbf{x},t)$ are time coarse-grained, i.e.

9 $$ \begin{aligned} E(\mathbf{x},t) &= \int_{-\infty}^{t} dt^{\prime} \alpha \bigl(t-t^{\prime}\bigr)n_{E} \bigl(\mathbf{x},t^{\prime}\bigr), \\ I(\mathbf{x},t) &= \int_{-\infty}^{t} dt^{\prime} \alpha \bigl(t-t^{\prime}\bigr)n_{I} \bigl(\mathbf{x},t^{\prime}\bigr) , \end{aligned} $$

where $n_{E}(\mathbf{x},t)$ and $n_{I}(\mathbf{x},t)$ are the proportions of excitatory and inhibitory neurons activated per unit time. It follows from Eq. (4) that $\alpha(t)$ acts as a low-pass filter, and therefore that $E(\mathbf{x},t)$ and $I(\mathbf{x},t)$ are low-pass filtered version of $n_{E} (\mathbf{x},t^{\prime})$ and $n_{I} (\mathbf{x},t^{\prime})$, respectively. The net effect of such a coarse-graining is to remove oscillatory components of neural population responses greater than 100 Hz.

### Attractor Dynamics

**Fig. 10:**
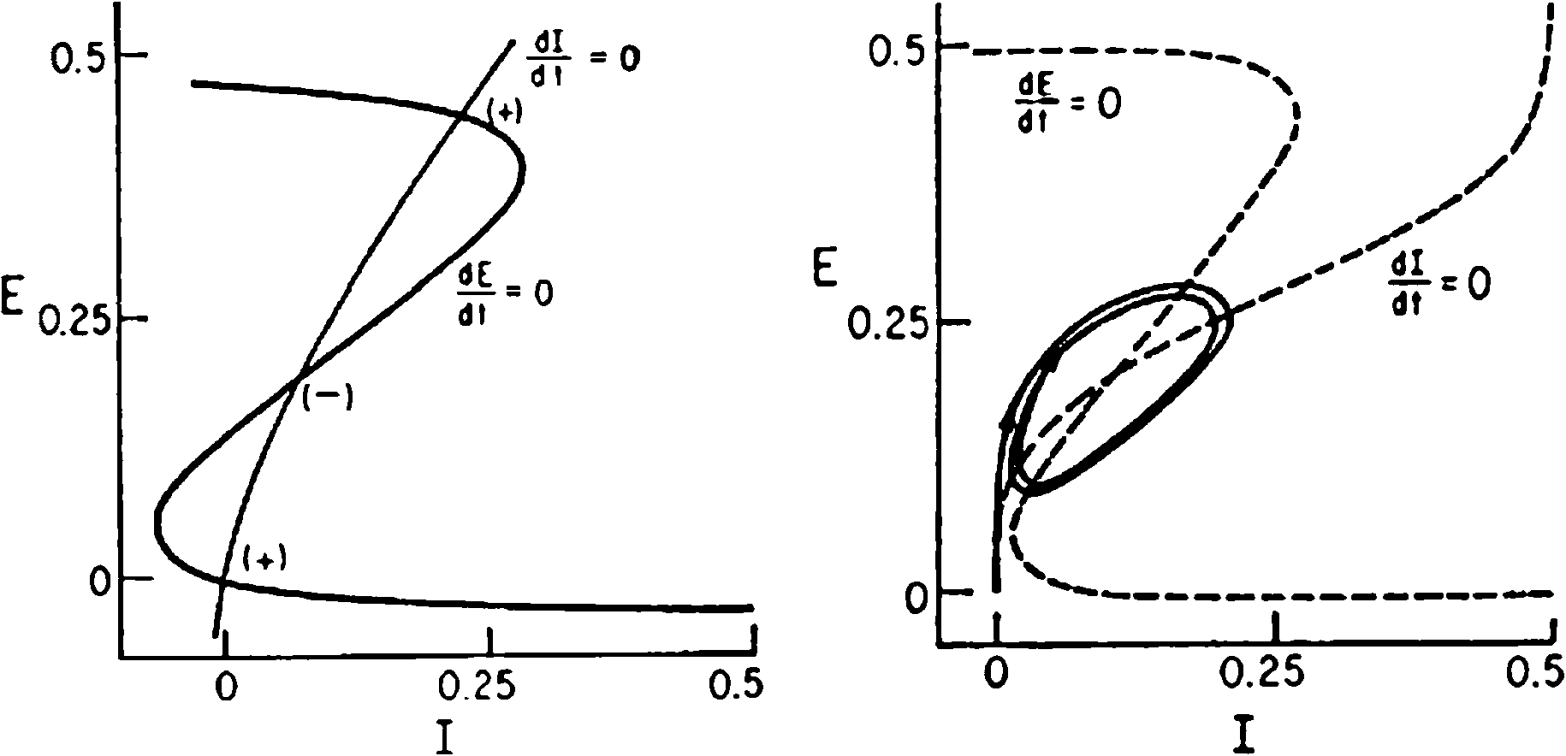
*The left panel* shows the *E*–*I* phase plane and nullclines of Eq. (7). The intersections of the two null clines are equilibrium or fixed points of the equations. Those labeled (+) are stable, those labeled (−) are unstable. Parameters: $w_{EE}=12$, $w_{EI}=4$, $w_{IE}=13$, $w_{II}=11$, $n_{H} = 0$. The stable fixed points are *nodes*. *The right panel* shows an equilibrium which is periodic in time. Parameters: $w_{EE}=16$, $w_{EI}=12$, $w_{IE}=15$, $w_{II}=3$, $n_{H}=1.25$. In this case the equilibrium is a *limit cycle*. [Redrawn from [24]]

A major feature of Eq. (7) is that it supports different kinds of asymptotically stable equilibria. Figure 10 shows two such equilibrium patterns: There is also another phase plane portrait in which the equilibrium is a damped oscillation, i.e., a stable *focus*. In fact by varying the synaptic weights $w_{EH}$ and $w_{IH}$ or $a=w_{EE} w_{II}$ and $b=w_{IE}w_{EI}$ we can move from one portrait to another. It turns out that there is a substantial literature dealing with the way in which such changes occur, The mathematical technique for analyzing these transformations is bifurcation theory, and it was first applied to neural problems 53 years ago by Fitzhugh [28], but first applied systematically by Ermentrout and Cowan [29–31] in a series of papers on the dynamics of the mean-field Wilson–Cowan equations. Subsequent studies by Borisyuk and Kirillov [32] and Hoppenstaedt and Izhikevich [33] have greatly extended this analysis.

**Fig. 11:**
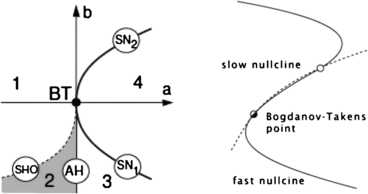
*The left panel* shows bifurcations of Eq. (7) in the spatially homogeneous case, organized around the Bogdanov–Takens (BT) bifurcation. *SN1* and *SN2* are saddle-node bifurcations. *AH* is an Andronov–Hopf bifurcation, and *SHO* is a saddle homoclinic-orbit bifurcation. Note that *a* and *b* are the control parameters introduced earlier. *The right panel* shows the nullcline structure of a Bogdanov–Takens bifurcation. At the Bogdanov–Takens point, a stable node (*open circle*) coalesces with an unstable point. [Redrawn from [34]]

The left panel of Fig. 11 shows the detailed structure around such bifurcations. Evidently the saddle-node and Andronov–Hopf bifurcations lie near the Bogdanov–Takens bifurcation. Thus all the bifurcations described in the spatially homogeneous Wilson–Cowan equations lie close to such a bifurcation in the $(\mathbf{a},\mathbf {b})$-plane. The Bogdanov–Takens bifurcation depends on two control parameters **a** and **b**, and is therefore of *codimension* 2. In such a bifurcation an equilibrium point can simultaneously become a marginally stable saddle and an Andronov–Hopf point. So at the bifurcation point the eigenvalues of its stability matrix have zero real parts. In addition the right panel of Fig. 11 shows how the fast E-nullcline and the slow I-nullcline intersect. The first point of contact of the two nullclines is the Bogdanov–Takens bifurcation point. The two nullclines remain close together over a large part of the subsequent *E*–*I* phase space before diverging. As we will later discuss, this property of the nullclines is closely connected with the existence of a *balance* between excitatory and inhibitory currents in the network described by the Wilson–Cowan equations, and therefore with the existence of *avalanches* in stochastic Wilson–Cowan equations [35].

## Stochastic Neural Dynamics

### Introduction

**Fig. 12:**
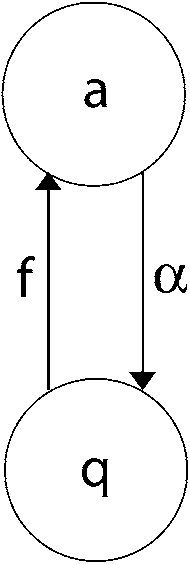
Neural state transitions. *a* is the activated state of a neuron. *q* is the quiescent state. *α* is a decay constant, but *f* depends on the number of activated neurons connected to the neuron, and on an external stimulus *h*

To develop such equations we need to reformulate neural population dynamics as a Markov process. We first consider the representation of the dynamics of a cortical sheet or slab comprising a single spatially homogeneous network of *N* excitatory binary neurons. Such neurons transition from a quiescent state *q* to an activated state *a* at the rate *f* and back again to the quiescent state *q* at the rate *α*, as shown in Fig. 12.

### A Master Equation for a Network of Excitatory Neurons

The first step is to formulate a master equation describing the evolution of the probability distribution of neural activity $P_{n} (t)$ in such a network. Consider first *n* activated neurons, each becoming quiescent at the rate *α*. This produces a flow out of the state *n* at rate *α*, proportional to $p_{n}(t)$, hence a term in the master equation of the form $-\alpha nP_{n}(t)$. Similarly the flow into *n* from the state $n+1$ produces a term $\alpha(n+1) P_{n+1}(t)$. The net effect is the term

10 $$ \alpha \bigl[ (n+1) P_{n+1}(t) - nP_{n}(t) \bigr]. $$

**Fig. 13:**
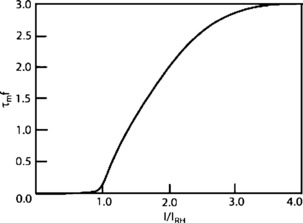
The firing rate function $f[s_{E} (n)]$, $\tau_{m} =1/\alpha= 3~\mbox{ms}$ is the neural membrane time constant, *I* is the input current, and $I_{\mathrm{RH}}$ is the *rheobase* or threshold current

Now consider the $N-n$ quiescent neurons in state *n*, each prepared to spike at rate $f[s_{E} (n)]$, leading to the term $-(N-n)f[s_{E} (n)] P_{n} (t)$, in which the total input is $s_{E} (n)=I(n)/I_{\mathrm{TH}} = (w_{EE} n + h_{E})/I_{\mathrm{TH}}$, and $f[s_{E} (n)]$ is the function shown in Fig. 13, a low-noise version of Eq. (3).

The flow into the state *n* from the state $n-1$ is therefore $(N-n+1)\times f[s_{E} (n-1)] P_{n-1} (t)$, and the total contribution from excitatory spikes is then

11 $$ (N-n+1)f\bigl[s_{E} (n-1) \bigr] P_{n-1} (t) - (N-n)f \bigl[s_{E} (n)\bigr] P_{n} (t). $$

It follows that the probability $P_{n} (t)$ evolves according to the master equation

12 $$\begin{aligned} \frac{dP_{n} (t)}{dt} =& \alpha \bigl[ (n+1) P_{n+1}(t) - nP_{n}(t) \bigr] \\ &{}+ (N-n+1)f\bigl[s_{E} (n-1) \bigr] P_{n-1} (t) - (N-n)f \bigl[s_{E} (n)\bigr] P_{n} (t). \end{aligned}$$

It is easy to derive an evolution equation for $\langle n(t) \rangle$, the average number of active neurons in the network, using standard methods. The equation takes the form

13 $$ \frac{d\langle n(t) \rangle}{dt} = -\alpha\bigl\langle n(t) \bigr\rangle + \bigl(N-\bigl\langle n(t) \bigr\rangle \bigr) f\bigl[\bigl\langle s_{E} (n) \bigr\rangle \bigr] , $$

where $\langle s_{E} (n) \rangle= w_{EE} \langle n \rangle+ h_{EE}$, and is the simplest form of Eq. (7) for a single excitatory population. Such a mean-field equation can be obtained in a number of different ways, in particular by using the van Kampen “system-size expansion” of Eq. (12) about a locally stable equilibrium [36]. However, as is well known, this expansion breaks down at a marginally stable critical point, e.g. at a Bogdanov–Takens point, and a different method must be used to analyze such a situation.

Before proceeding we note that these equations can be extended to cover the situation introduced in Eq. (7) which incorporates spatial effects. The variable $n(t)/N$ is extended to $n(\mathbf{x},t)$ representing the *density* of active neurons at the cortical location **x** at time *t*, and the total input current $I(n)$ becomes the current density

14 $$ I \bigl(n(\mathbf{x})\bigr) = \int d^{d} x^{\prime} w_{EE} \bigl(\mathbf{x} - \mathbf {x}'\bigr) n\bigl(\mathbf{x}'\bigr) + h_{E} (\mathbf{x}) . $$

### A Master Equation for a Network of Excitatory and Inhibitory Neurons

Since about $1/5$th of all cortical neurons are inhibitory, it is important to include the effects of such inhibition. We therefore extend Eq. (10) to include inhibitory neurons. The result is the master equation:

15 $$\begin{aligned} \frac{dP(n_{E}, n_{I},t)}{dt} =&\alpha_{E} \bigl[(n_{E} + 1)P(n_{E} + 1, n_{I},t) -n_{E} P(n_{E},n_{I},t) \bigr] \\ &{}+ \bigl[ (N_{E} -n_{E} + 1) f_{E} \bigl[s_{E} (n_{E} -1,n_{I})\bigr]P(n_{E}-1,n_{I},t) \\ &{}- (N_{E} - n_{E}) f_{E} \bigl[s_{E} (n_{E}, n_{I})\bigr]P(n_{E}, n_{I},t) \bigr] \\ &{}+ \alpha_{I} \bigl[(n_{I} +1) P(n_{E},n_{I} +1,t) -n_{I} P(n_{E}, n_{I},t) \bigr] \\ &{}+ \bigl[ (N_{I} -n_{I} + 1) f_{I} \bigl[s_{I} (n_{E},n_{I}-1)\bigr]P(n_{E},n_{I}-1,t) \\ &{}- (N_{I} - n_{I}) f_{I} \bigl[s_{I} (n_{E}, n_{I})\bigr]P(n_{E}, n_{I},t) \bigr] . \end{aligned}$$

See Benayoun et al. [35] for a derivation of this equation. It is easy to derive Eq. (7) from this master equation. However, there is much more information as regards stochastic neural dynamics contained in Eq. (15) than is contained in such an equation. We refer, of course, to the effects of intrinsic fluctuations and of correlations.

## Analyzing Intrinsic Fluctuations

To analyze such effects we need to look more closely at the attractor dynamics of Eq. (7). There are two cases to consider. In case 1, the attractor is either an asymptotically stable node or focus, or else a limit cycle. In case 2, the attractor is only marginally stable. In nonlinear dynamics this is a bifurcation point, e.g. a Bogdanov–Takens point, or a saddle node or Andronov–Hopf point. In statistical mechanics this is the critical point of a phase transition.

### The System-Size Expansion

The system-size expansion was introduced by van Kampen [36] to analyze the effects of intrinsic fluctuations in case 1. The intuition behind this approach comes from the idea that if neurons are independently activated, then the total activity in a excitatory neural network in such a case is Gaussian distributed, with mean activity $\langle n_{E} (t) \rangle$ proportional to *N*, the total number of neurons in the network, and standard distribution proportional to $\sqrt {N}$. So the number of neurons activated at a given time can be represented by the variable

16 $$ k = N n_{E} + \sqrt{N} \xi_{E} , $$

where $\xi_{E}$ is a Gaussian random perturbation.

The deterministic term satisfies Eq. (7), the random variable satisfies the linear Langevin equation

17 $$ \frac{d \xi_{E}}{dt} = A\xi_{E} + \sqrt{\alpha_{E} n_{E} + (1-n_{E})f_{E}\bigl[s_{E}(n_{E}) \bigr]} \eta_{E} $$

to order $N^{-1/2}$, where *A* is a constant and $\eta_{E}$ is an independent white noise variable, whose amplitudes are calculated from Eq. (7).

**Fig. 14:**
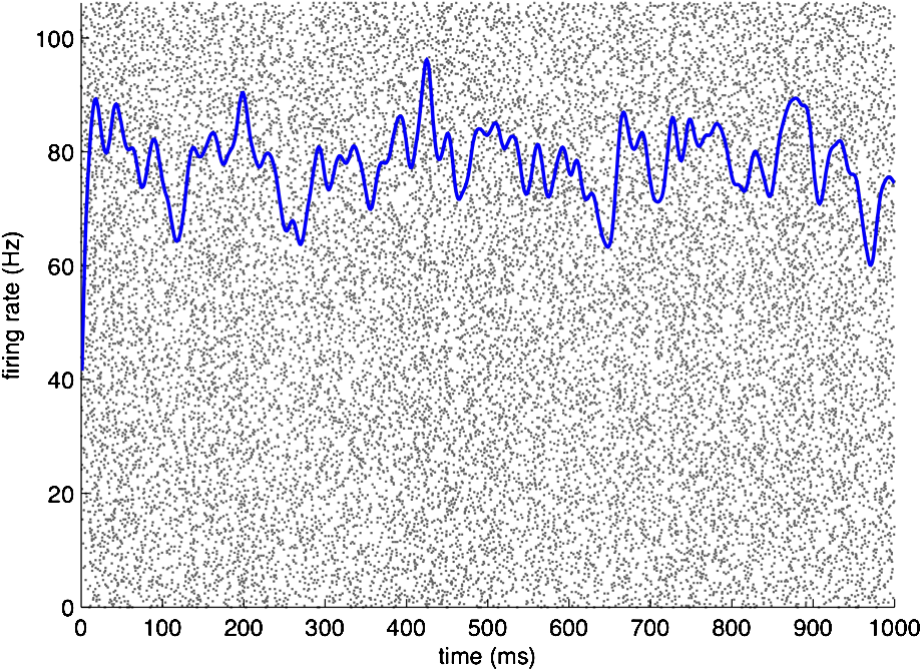
Raster plot of the spiking patterns in a network of $N=800$ excitatory neurons. *Each black dot* represents a neural spike. The mean activity $\langle n_{E} (t) \rangle$ is represented by the *blue trace*. Simulation using the Gillespie algorithm with parameter values $h_{E}=h_{I}=0.001$, $w_{0} =w_{E}-w_{I} = 0.2$, and $w_{E} + w_{I} = 0.8$. [Redrawn from [35]]

**Fig. 15:**
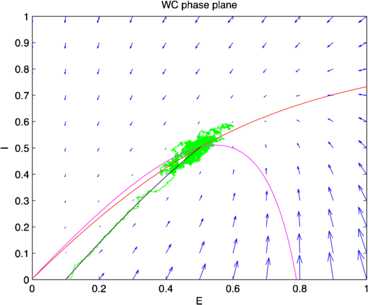
Phase plane plots of the activity shown in Fig. 14 showing the vector field (*blue*) and nullclines $\dot{E}=0$ (*magenta*) and $\dot{I}=0$ (*red*), of Eq. (1) and plots of a deterministic (*black*) and a stochastic (*green*) trajectory starting from identical initial conditions. [Redrawn from [35]]

**Fig. 16:**
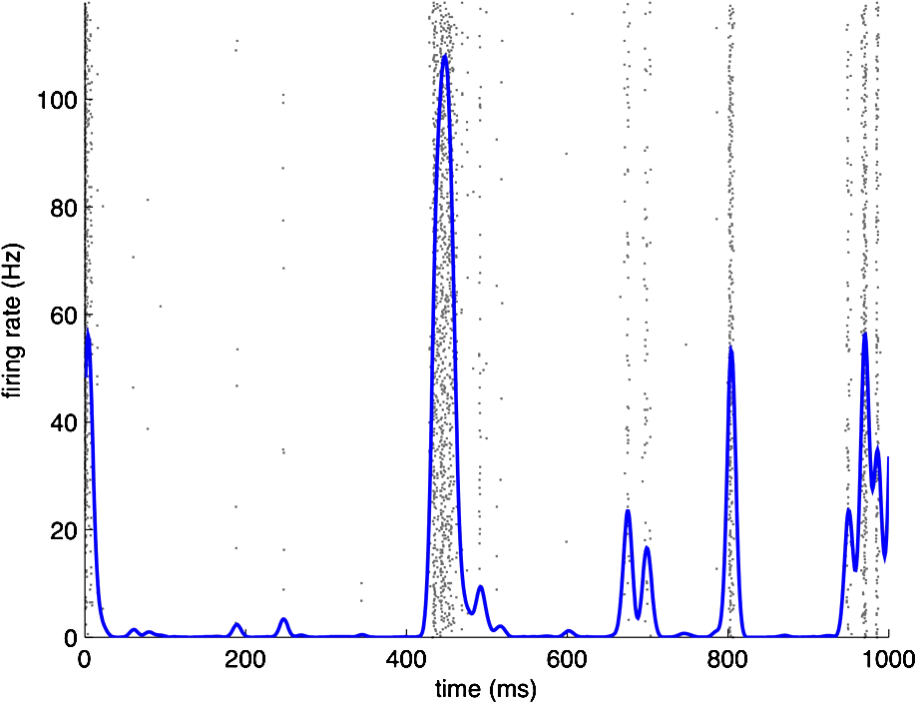
Raster plot of the spiking patterns in a network of $N=800$ excitatory neurons. *Each black dot* represents a neural spike. The mean activity $\langle n_{E} (t) \rangle$ is represented by the *blue trace*. Simulation using the Gillespie algorithm with parameter values $h_{E}=h_{I}=0.001$, $w_{0} =w_{E}-w_{I} = 0.2$, and $w_{E} + w_{I} = 13.8$. [Redrawn from [35]]

**Fig. 17:**
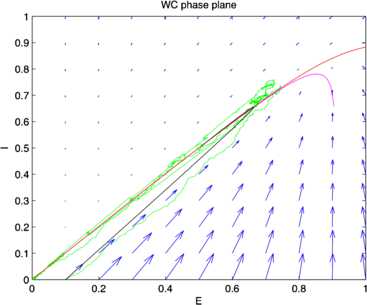
Phase plane plots of the activity shown in Fig. 16 showing the vector field (*blue*) and nullclines $\dot{E}=0$ (*magenta*) and $\dot{I}=0$ (*red*), of Eq. (1) and plots of a deterministic (*black*) and a stochastic (*green*) trajectory starting from identical initial conditions. [Redrawn from [35]]

An early version of this application of the system-size expansion can be found in Ohira and Cowan [37]. The extension to the excitatory and inhibitory neural network introduced in Eq. (7) is to be found in Benayoun et al. [35]. This paper is notable for its use of the Gillespie algorithm [38]. In this algorithm the simulation time is advanced only when the network’s state is updated, and the time intervals *dt* are random variables dependent upon the network state. The simulation is carried out for a network in which certain symmetry conditions are introduced. These conditions are

18 $$ w_{IE} = w_{EE} = w_{E};\quad\quad w_{EI} = w_{II} = w_{I};\quad\quad w_{E} - w_{I} = w_{0} , $$

where $w_{0}$ is kept constant. Figures 14, 15, 16, 17 show the results.

**Fig. 18:**
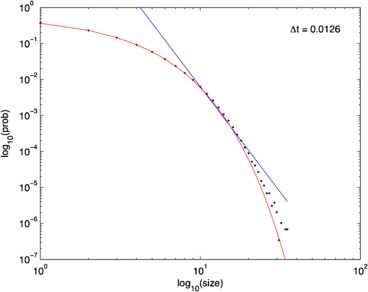
Network burst distribution in number of spikes, together with geometric (*red*) and power law (*blue*) fit; Δ*t*, the mean inter-spike interval, is the time bin used to calculate the distribution, and $\beta=-1.62$ is the slope exponent of the fit. Simulation using the Gillespie algorithm with parameter values $h_{E}=h_{I}=0.001$, $w_{0} =w_{E}-w_{I} = 0.2$, and $w_{E} + w_{I} = 0.8$. [Redrawn from [35]]

**Fig. 19:**
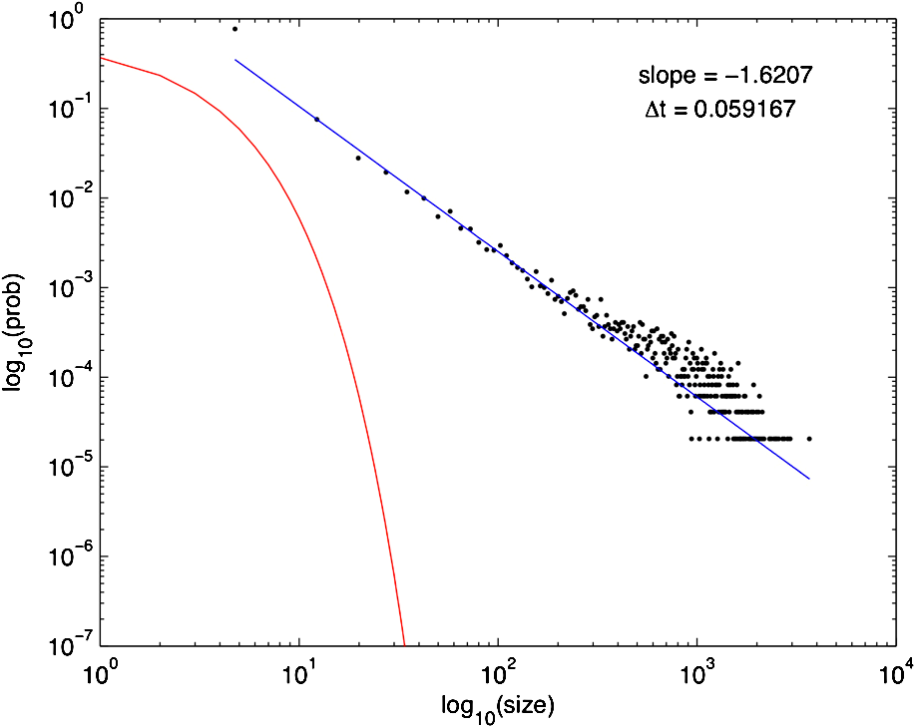
Network burst distribution in number of spikes, together with geometric (*red*) and power law (*blue*) fit; Δ*t*, the mean inter-spike interval, is the time bin used to calculate the distribution, and *β* is the slope exponent of the fit. Simulation using the Gillespie algorithm with parameter values $h_{E}=h_{I}=0.001$, $w_{0} =w_{E}-w_{I} = 0.2$, and $w_{E} + w_{I} = 13.8$. [Redrawn from [35]]

It should be evident from a study of these figures that the location of the fixed point of Eq. (7) remains unchanged as $w_{E} + w_{I}$ increases from 0.8 to 13.8, but the stochastic trajectory (green) becomes increasingly spread out as the nullclines become more parallel. Such a feature is also evident in the right panel of Fig. 11 in which the nullcline structure of the Bogdanov–Takens bifurcation is shown. It is also evident that a qualitative change has taken place in the nature of the activity: it has changed from random fluctuations to random bursts. Figures 18 and 19 make this clear.

### Symmetries and Power Laws

It will be seen that the simulations described above, in which the network symmetry represented in Eq. (17) is present, have uncovered an important property, namely that a stochastic version of Eq. (7) incorporating such a symmetry can spontaneously generate random activity in the form of bursts, whose statistical distribution is a power law. The other important property concerns the basic network dynamics generating such bursts.

We first note the experimental data provided by DeLisle Burns [7] and Beggs and Plenz [9] described in the introduction, and then we discuss the underlying neurodynamics. The main result of the Beggs–Plenz observations is that isolated slices generate bursting behavior similar to that found in the simulations, with a power law burst distribution with slope exponent of $\beta= -1.5$. This should be compared with the simulation data shown in Fig. 18 in which $\beta=-1.62$. Note, however, that the geometry of our network simulation is not comparable with that of a cortical slice. It remains to carry out simulations of the stochastic version of Eq. (7) on a 2-dimensional lattice. Work on this is currently ongoing. In any event, the Beggs–Plenz paper generated a great deal of interest in the possibility of critical behavior in the sense of statistical physics existing in stochastic neural dynamics, including the possibility that brain dynamics exhibits self-organized criticality. In the later parts of this paper, we briefly address this possibility.

#### Random Bursting

We turn now to the neuro-dynamics underlying random bursting. We first note that the fixed point of the dynamics remains unchanged as $w_{E} + w_{I}$ increases from ${0.8 \rightarrow13.8}$, and $n_{E} = n_{I}$. We also recall by Eq. (18) that $w_{E}-w_{I} = w_{0} = 0.2$, so that as the network begins to fire in random bursts,

19 $$ w_{0} \ll w_{E} + w_{I}. $$

This inequality has a number of consequences [35, 39]. Most importantly, it allows a particular change of variables in Eq. (12) extended to include inhibition.

20 $$ \begin{aligned} \frac{d\langle n_{E} (t) \rangle}{dt} &= -\alpha\bigl\langle n_{E} (t) \bigr\rangle + \bigl(1-\bigl\langle n_{E}(t) \bigr\rangle \bigr) f\bigl[\langle s \rangle\bigr], \\ \frac{d\langle n_{I} (t) \rangle}{dt} &= -\alpha\bigl\langle n_{I} (t) \bigr\rangle + \bigl(1-\bigl\langle n_{I}(t) \bigr\rangle \bigr) f\bigl[\langle s \rangle\bigr], \end{aligned} $$

where $\langle s \rangle= w_{E} n_{E} - w_{I} n_{I} + h$, and $\langle n_{E} \rangle$ and $\langle n_{I} \rangle$ are interpreted as the mean fractions of activated neurons in the network.

Now introduce the change of variables

21 $$ \varSigma= \frac{1}{2} (n_{E} + n_{I}), \quad\quad\Delta= \frac{1}{2} (n_{E} - n_{I}), $$

so that Eq. (20) transforms into the equation

22 $$ \begin{aligned} \frac{d\langle\varSigma(t) \rangle}{dt} &= -\alpha\bigl\langle \varSigma(t) \bigr\rangle + \bigl(1-\bigl\langle \varSigma(t) \bigr\rangle \bigr) f\bigl[\langle s \rangle\bigr], \\ \frac{d\langle\Delta(t) \rangle}{dt} &= - \bigl\langle \Delta(t) \bigr\rangle \bigl(\alpha+ f \bigl[\langle s \rangle\bigr] \bigr). \end{aligned} $$

Such a transformation was introduced into neural dynamics by Murphy and Millar [39], and used by Benayoun et al. [35]. But it was introduced much earlier by Janssen [40] in a study of the statistical mechanics of stochastic Lotka–Volterra population equations on lattices, which are known to be closely related to stochastic neural population equations on lattices [41].

The important point about the transformed equations is that they are *decoupled*, with the unique stable solution $(\varSigma_{0}, 0)$, which is equivalent to $n_{E} = n_{I}$ in the original variables. This is precisely the stable fixed point used in the simulations. Note also that, in the new variables *Σ* and Δ, the fixed point current is

23 $$ s = w_{0} \varSigma+ (w_{E} + w_{I}) \Delta+ h. $$

So at the stable fixed point $(\varSigma_{0}, 0)$, $s = w_{0} \varSigma_{0} + h$. Near such a fixed point, Δ is only weakly sensitive to changes in *Σ*, and $\varSigma_{0}$ is unchanged when varying $w_{E} + w_{I}$ for constant $w_{0}$. Murphy and Miller called Eq. (20) an *effective feed-forward system* exhibiting a balance between excitatory and inhibitory currents, and a *balanced amplification* of a stimulus *h*.

We can now perform a system-size expansion of the associated master equations [35], to obtain a two component linear Langevin equation for small Gaussian fluctuations about the stable fixed point $(\varSigma_{0}, 0)$. This takes the form

24 $$ \frac{d}{dt} \begin{pmatrix} \xi_{\varSigma}\\ \xi_{\Delta}\end{pmatrix} = \begin{pmatrix} -\lambda_{1} & w_{\mathrm{ff}} \\ 0 & -\lambda_{2} \end{pmatrix} \begin{pmatrix} \xi_{\varSigma}\\ \xi_{\Delta}\end{pmatrix} +\sqrt{\alpha \varSigma_{0}}\begin{pmatrix} \eta_{\varSigma}\\ \eta_{\Delta}\end{pmatrix}, $$

where the eigenvalues are $\lambda_{1} = (\alpha+f[s_{0}])+(1-\varSigma_{0})w_{0} f'[s_{0}]$ and $\lambda_{2}=(\alpha+f[s_{0}])$, and $w_{\mathrm{ff}}=(1-\varSigma _{0})(w_{E} + w_{I})f'[s_{0}]$.

The Jacobian matrix

$$A= \begin{pmatrix} -\lambda_{1} & w_{\mathrm{ff}} \\ 0 & -\lambda_{2} \end{pmatrix} $$

is upper triangular and has eigenvalues $-\lambda_{1}$ and $-\lambda_{2}$. It follows that when $w_{0}$ is small and positive, then so are the eigenvalue magnitudes $\lambda_{1}$ and $\lambda_{2}$. So the eigenvalues are small and negative and the fixed point $(\varSigma_{0},0)$ is weakly stable. Evidently *A* lies close to the matrix

$$B = \begin{pmatrix} 0 & w_{\mathrm{ff}} \\ 0 & 0 \end{pmatrix} = \begin{pmatrix} 0 & 1 \\ 0 & 0 \end{pmatrix} w_{\mathrm{ff}}= \bar{B} w_{\mathrm{ff}}. $$

But the matrix *B̄* is the signature of the *normal form* of the Bogdanov–Takens bifurcation [33]. Thus the weakly stable node lies close to a Bogdanov–Takens bifurcation, as we have suggested.

### Intrinsic Fluctuations at a Marginally Stable Fixed Point

We now turn to case 2, in which the network dynamics is at a marginally stable fixed point. As we showed earlier, such a fixed point is a Bogdanov–Takens point. We cannot use the system-size expansion at such a point, but we can use the methodology and formalism of statistical field theory [42–45]. However, for the neuro-dynamics considered in this article, case 1 applies: the resting and driven activities are all at or near a weakly stable fixed point. Despite this, the fact that the fixed point is only weakly stable indicates that the resting and weakly driven states lie in what has been called the *fluctuation-driven* region near the marginally stable fixed point [46]. Thus we need to outline some of the results of the analysis of case 2. The reader is referred to the details in the article by Cowan et al. [45].

The basic result is that the stochastic equivalent of the Bogdanov–Takens bifurcation is the critical point of a *Directed Percolation* phase transition, or DP [47]. In DP there are two stable states, separated by a marginally stable critical point. One of these is an *absorbing* state, corresponding to the neural population state in which all neurons are quiescent, so that the mean number of activated states or *order parameter*$\langle n \rangle = 0$. The other is one in which many neurons are activated, so that $\langle n \rangle\neq0$ in the *activated* state. At a critical point the quiescent state becomes marginally stable and is driven by fluctuations into the activated state.

What is important for the present study is that in the neighborhood of such a critical point, i.e. in the fluctuation-driven regime, there are two significant features of the activity which relate to the experimental data we have described: (a) the resting behavior shows random burst behavior whose statistical signature is consistent with DP, i.e., the distribution of bursts follows a power law with slope exponent −1.5, which is the slope of several forms of random percolation, including what is called mean-field DP [9, 10]; (b) intrinsic *correlations* are large, and pair correlations extend over significant cortical distances [18].

## Modeling the Experimental Data

### Resting Activity

#### Random Burst Activity

Assuming that the resting state occurs in the neighborhood of a weakly stable node or focus, to start with we can use the results of the system-size expansion of the *E*–*I* master equation described earlier. The conclusion we reach is that in the case that there is a *balance* between excitation and inhibition, so that the network is at weakly stable node, or possible a focus, then random burst behavior with a power law slope exponent close to −1.5 is seen [35]. This is the result shown in Figs. 14–19, and of course the result is also completely consistent with the Beggs–Plenz data plotted in Figs. 5 and 6. We also note that these results are completely consistent with our recent analysis, Cowan [45], and with recent experimental data that demonstrates the sub-criticality of the resting state by Priesemann et al. [48].

#### Pair Correlations

As to pair correlations associated with resting or spontaneous activity, we refer to Fig. 9 in which the measured resting pair correlation falls off with pair separation, in both cats and monkeys. This finding can be replicated within the theoretical framework we have established in two differing ways.

**Fig. 20:**
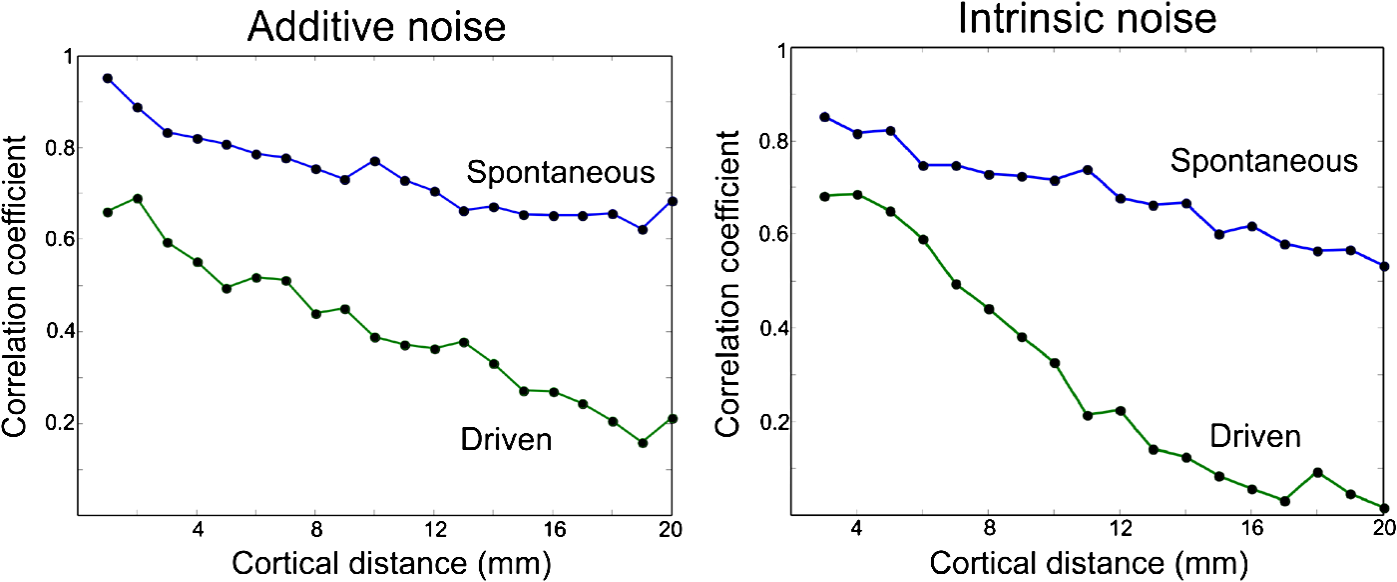
*The left panel* shows the pair-correlation function for resting and driven activity, for additive Gaussian noise, *the right panel* that for resting and driven activity, for intrinsic noise, averaged over many simulations using the Gillespie algorithm. [Reproduced from [38]]

(a) We first make use of Eq. (7), the mean-field Wilson–Cowan equations for the 1D-spatial case, and simply add *δ*-correlated Gaussian noise to the equations. The resulting pair-correlation function for resting activity is shown in the left panel of Fig. 20. (b) We then use the stochastic Wilson–Cowan master equation introduced in Eq. (14), extended to the spatial case. In such a case the noise is multiplicative and intrinsic, and we used the Gillespie algorithm [38] to simulate the process.

Such simulations of the behavior of Wilson–Cowan equations replicate very accurately, the pair-correlation behavior shown in Fig. 9, reported in [13], both for resting activity and for driven activity.

### Driven Activity

#### Weak Stimuli

**Fig. 21:**
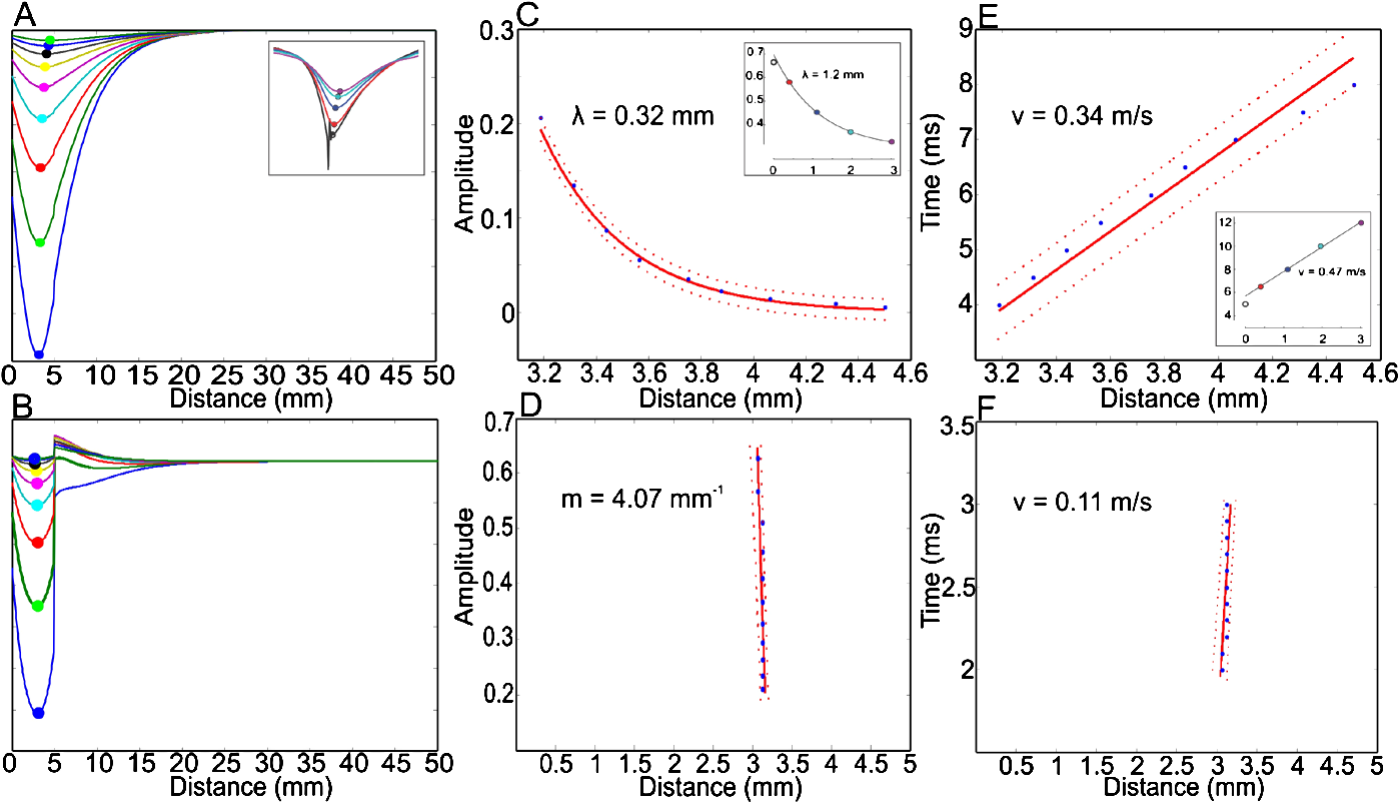
**A** Variation in the LFP amplitude of decaying waves. The largest amplitude is the initial response to a brief weak current pulse. **B** The exponential decay of the LFP amplitude, as a function of distance traveled. **C** Time–distance plot of the peak amplitude indicating that the velocity of wave propagation is constant at about $0.3~\mbox{m}\, \mbox{s}^{-1}$. **D** Localized LFP in response to a strong current pulse. **E** Rapid decay of the amplitude in a linear fashion. **F** Very slow propagation of the LFP

We now consider the results reported by Carandini et al. [12–14], of traveling, decaying waves seen in LFP, shown in Figs. 7 and 8; and by Muller and Destexhe [15], in VSD recordings, in response to brief weak current pulses. These results can be replicated quite precisely in simulations of Eq. (8), in which the network dynamics is near the balanced state in which $E \approx I$. The top row of Fig. 21 shows a simulation of these simulations. These results should be compared with those plotted in Fig. 7. It should be clear that the simulations replicate very accurately, such data.

#### Strong Stimuli

The other result reported by Carandini et al. is that for strong stimuli the resulting LFP does *not* propagate very far and remains localized. This property was actually reported in Wilson and Cowan’s 1973 paper [27]! The bottom row of Fig. 21 shows a current simulation of this property, again in which the network state is approximately balanced.

### Explaining the Differing Effects of Weak and Strong Stimuli

It is evident that there are big differences between the effects produced by weak and strong stimuli. What is the cause of such differences? Given that the only parameter in the Wilson–Cowan equations that is varied in the two cases is the stimulus intensity, this suggests that the property which causes the different responses is the level of inhibition. It must therefore be the case that the threshold for inhibitory activity is set high enough that weak stimuli do not trigger inhibitory effects, whereas strong enough stimuli do trigger such effects. Indeed this is one of the possibilities suggested by Carandini et al. in their papers. Thus inhibition blocks LFP (and VSD) propagation.

This possibility is also consistent with the effects of stimuli on pair correlations. We predict that the pair-correlation function should falloff more slowly in the case of resting or weakly driven activity, than in the case of stronger stimuli. Such a result would be consistent with the suggestions of Churchland et al. that one effect of stimuli is to lower noise levels.

## Discussion

### Early Work

The main results described in this article concern the use of the Wilson–Cowan equations to analyze the dynamics of large populations of interconnected neurons. Early workers, including Shimbel and Rapaport [20] and Beurle [21], appreciated the need to use a statistical formulation of such dynamics, but lacked the techniques to go beyond mean-field theory. The Wilson–Cowan equations [24, 27] were the first major attempt at a statistical theory, but still lacked a treatment of second and higher moments. However, what the equations did describe was mathematical conditions for *attractor* dynamics. Further work by Ermentrout and Cowan [29–31] and by Borisyuk and Kirillov [32], and Hoppenstaedt and Izhikevich [33, 34] used the mathematical techniques of bifurcation theory to more fully analysis such dynamics. The main result was that neural population dynamics is organized around a Bogdanov–Takens bifurcation point, in the neighborhood of which (in a phase space of two control parameters) are saddle-node and Andronov–Hopf bifurcations. Thus neural network dynamics contains locally stable equilibria in the form of stationary and oscillatory attractors.

### The System-Size Expansion

The problem of going beyond the mean-field regime proved to be very difficult. Some progress was made by Ohira and Cowan [37] formulating stochastic neural dynamics in the neighborhood of a stable stationary equilibrium as a random Markov process and using the Van Kampen system-size expansion [36]. Further process along these lines was made by Benayoun et al. [35] who formulated Eq. (7) as a random Markov process. But Benayoun et al. went further, by incorporating some symmetries into Eq. (7) discovered by Murphy and Miller [39] which, in retrospect, located the stationary equilibrium of the equations near a Bogdanov–Takens point. The result was that the stochastic version of Eq. (7) generates the random bursts of activity we now refer to as *avalanches*. In addition the avalanche distribution was that of a power law, with a slope exponent $\beta= 1.6$. This value is close to that observed by Beggs and Plenz [9] in their observations of neural activity in an isolated cortical slab, of avalanche distributions with a slope exponent of $\beta= 1.5$.

### A Statistical Theory of Neural Fluctuations

There remained the problem of developing a statistical theory for the fluctuations about a marginally stable critical point, such as a Bogdanov–Takens point. This problem was formulated by Cowan [42] and solved by Buice and Cowan [43, 44]. This is a major result since it connects the theory of stochastic neural populations at a critical point, with many well studied examples of other populations of interconnected units. Examples include percolation in random graphs, branching and annihilating random walks, catalytic reactions, interacting particles, contact processes, nuclear physics, and bacterial colonies. Many of these processes are subject to a phase transition, known as a directed percolation phase transition (DP). and all these processes have the same statistical properties, including the appearance of random bursts or avalanches.

### Relation to Experimental Data

However, although the statistical theory is relevant to the pair-correlation problem, it is the mean-field Wilson–Cowan equations that proved to be necessary and sufficient to analyze neocortical responses to brief stimuli, both weak and strong. In our opinion the close fit between the data and the simulations of the Wilson–Cowan equations with fixed parameters is quite remarkable, especially given the fact that these equations were formulated some 45 to 50 years ago! More detailed papers dealing with these and other results on neocortical responses to stimuli are in preparation.
